# Optical Emission Spectroscopy on the Gaseous Electronics Conference RF Reference Cell

**DOI:** 10.6028/jres.100.027

**Published:** 1995

**Authors:** J. R. Roberts

**Affiliations:** National Institute of Standards and Technology, Gaithersburg, MD 20899-0001

**Keywords:** Gaseous Electronics Conference, optical emission, plasma, reference cell, rf discharge, spatial, spectroscopy, temporal

## Abstract

A summary of the experimental observations of the optical emission from the Gaseous Electronics Conference (GEC) rf Reference Cell plasma will be discussed. Spatially and temporally resolved results are provided for various reference and non-reference conditions, including etching type plasmas. These measurements provide a detailed description of the temporal evolution of optical emission from different excited atomic states within different atomic and ionic species, as well as their radial and axial distributions. Some of the measurements have been placed on a absolute radiometric scale to provide comparisons to model calculations. Spectral line profile measurements are also presented to provide some insight into the multi-component particle velocities present in such plasmas.

## 1. Introduction

Optical emission spectroscopy (OES) provides a non-evasive probe to investigate atoms, ions and molecules within a plasma. It can provide information about properties, such as (excited state) species densities, electronatom, atom-atom and ion-atom collisional effects, energy distribution of species, charge transfer between plasma constituents, and electric and magnetic fields to name a few. Its use as a diagnostic tool for emitting media has led to a greater understanding of very complex phenomena such as the evolution of stellar atmospheres and the study of fusion plasmas. Because the interpretation of OES observations is dependent on the source of emission and the understanding of the physical processes occurring within the source, care must be taken in the interpretation of these observations, e.g., see Ref. [1] and Ref. [2]. The use of OES in the diagnosis of low density, low temperature plasmas has been ubiquitous and has yielded a great deal of information about the properties of materials within the plasma. Its application in plasmas that are appropriate for processing of materials, such as semiconductor etching, is widespread and has been used, along with other diagnostics, to direct researchers and engineers in a pursuit of ever smaller features in semiconductor devices. Examples of the use of OES to understand the plasma processing of semiconducting materials can be found in Ref. [3].

### 1.1 OES on the Gaseous Electronics Conference (GEC) RF Reference Cell

The topics to be discussed in this paper will be limited to those that deal with OES as applied to the GEC rf Reference Cell [4]. Other optical measurement methods pertaining to the reference cell, such as laser induced fluorescence, are presented in articles within the present journal volume. The topics to be discussed here include: OES measurements concepts, details of OES experiments, spatially and temporally resolved excited state atom and ion distributions; the correlation of semiconductor material etch rates with radial atomic distributions; the use of OES as a teaching tool; the use of Abel inversion algorithms to invert OES line-of-site measurements and their comparison with laser induced fluorescence; and studies of multiple velocity distributions of atoms within the reference cell.

Some of the results presented here use the reference cell as an experimental platform with gases other than the reference gas, argon, e.g., helium, oxygen, nitrogen and etching gases as well as non-reference conditions. This demonstrates the cell as a useful and versatile device. Also, the optical emission results have been obtained along with other diagnostic techniques, such as ion energy analyzed mass spectrometry observations and detailed electrical measurements. This is done in an attempt to find correlations between various measurement methods.

### 1.2 Characteristics of the GEC RF Reference Cell

The GEC rf Reference Cell is described in detail in Ref. [4] as well as in Ref. [5]. The physical and electrical characteristics that are presented in these references define this experimental platform as a system for the study of asymmetrically driven, rf powered, parallel plate plasma discharges. The cylindrical vacuum chamber is constructed of stainless steel and has eight radial copper-gasketed flanges centered at the electrode midplane. Two 203 mm diameter flanges are fitted with 136 mm diameter quartz windows for OES observations. This allows the emission from the discharge to be observed beyond the 102 mm diameter electrodes throughout the ultraviolet, visible and infrared regions of the spectrum.

## 2. OES Measurement Concepts

In this section the concepts that are utilized in making OES studies of any emitting or absorbing medium are reviewed as well as the method of quantifying these measurements so absolute comparisons can be made in OES measurements and models.

### 2.1 Fundamental Concepts [6,7]

If we assume there is an atomic medium in a radiation field, and that the energy of the medium is affected by collisions of particles (atoms, ions, molecules, electrons) and the radiation field itself, through spontaneous emission, absorption and induced emission (negative absorption), then over a length d*s*, the equation governing the radiative transfer can be written as

$$dI_{\lambda }/ds=\left[n_{u}\;A_{\text{ul}}-I_{\lambda }\left(n_{l}B_{\text{lu}}-n_{u}B_{\text{ul}}\right)\right]h\;c\;P\left(\lambda \right)/\lambda$$ (1)

where *I_λ_* is the radiant power per unit area per unit wavelength-band (spectral irradiance), *s* is the emission length, *n*_u_ and *n*_l_ represent the upper and lower state population densities of the atomic medium respectively, *A*_ul_, *B*_ul_, and *B*_lu_ are the atomic state’s Einstein coefficients for emission, induced emission and absorption respectively [8,9], *h* is Plank’s constant, *c* is the velocity of light, *λ* is the emission wavelength and *P*(*λ*) is the spectral line shape factor. The integral of *P*(*λ*) over all wavelengths is defined equal to 1. *B*_ul_ and *B*_lu_ are related by their respective statistical weights *g*_u_ and *g*_l_ [6] as follows, *g*_u_ × *B*_ul_ = *g*_l_ × *B*_lu_. In the case of many discharges the density of the lower states are usually significantly larger than the upper states because of the relative low temperature of the medium or the lower state is metastable. In the case of the reference cell discharge, this is generally true of the excited states that are not metastable. This means that the solution of Eq. (1) can be written as

$$I_{\lambda }=I_{\lambda }\left(s=0\right)e^{-k_{\lambda }s}+n_{u}\;A_{\text{ul}}\;h\;c/\lambda \;k_{\lambda }\left[1-e^{-k_{\lambda }s}\right]$$ (2)

where *k_λ_* is the atomic transition’s absorption coefficient and

$$k_{\lambda }\equiv n_{l}\;B_{\text{lu}}\;h\;c\;P\left(\lambda \right)/\lambda .$$ (3)

When the upper state density (× *B*_ul_) is of the same order as the lower state density (× *B*_lu_), the general solution of Eq. (1) must be used to define the radiative transfer and must include induced emission. Under these conditions the medium could be so reabsorbing that interpretation of the OES signal would be difficult [1].

For OES observations in the reference cell, usually there is no external radiation field so *I_λ_* (*s* = 0) = 0. For an optically thin medium, i.e., *k_λ_s* ≪ 1 and no external radiation field, Eq. (2) can be reduced to

$$I_{\lambda }\approx n_{u}\;A_{\text{ul}}\;h\;c\;s/\lambda .$$ (4)

There are cases where there is radiation from outside the emitting atomic medium, therefore *I_λ_* (*s* = 0) ≠ 0. An example of this would be an external source, such as a lamp or a laser, directed along the line-of-sight of the observation to make absorption measurements within the medium. If this source has a known spectral irradiance, i.e., a calibrated source, then it can be used to determine absolute population densities of the lower atomic states of the emitting medium. Another example of an application where there is an external radiation field is the use of a concave spherical mirror positioned behind the emitting medium, along the line-of-sight, to reflect the emitting medium’s own radiation field back onto itself. This example is typically used to measure the medium’s optical depth, *k_λ_s*.

### 2.2 Radiance Calibration

It is important to define the quantities involved in the use of OES as a diagnostic tool. We normally deal with volume emitters when observing OES signals, e.g., the reference cell has volume between its electrodes of approximately 206 cm^3^. A portion of this volume emission is focused onto the slit of a spectrometer via a lens or mirror optical system. The calibration of the detected signals are normally done by substituting a *surface emitting* continuum source of known spectral radiance, like a calibrated tungsten strip lamp, in place of the volume emitting source. This calibrated lamp is positioned at the focus of the emission optical system and the amount of its radiation per unit time per unit solid angle per unit wavelength-band per unit area element is accurately known. Thus, the signal from the spectrometer and detection system is calibrated [10]. The spectral radiance of any emitter, *L_λ_*, is given in units of radiant energy per unit time per unit area per unit solid angle per unit wavelength-band, e.g., watts per sec per cm^2^ per steradian per nm.

$$L_{\lambda }=E_{\lambda }/\left(dA\;d\omega \;d\lambda \right).$$ (5)

If this spectal radiance is integrated over its wavelength-band, one obtains the total spectral radiance or integrated radiance, *L* in watts per cm^2^ per steradian.

$$L=\int L_{\lambda }\;d\lambda .$$ (6)

The realization of the calibration of the radiance of a spectral line emitted from a volume source using a surface emitting continuum source requires some clarification. Most often in the case of low temperature, low density plasmas the bandwidth of the spectral line is small compared to the bandwidth of the dispersive device that is used in the observations. This will happen in different ways. When the entrance slit width of the spectrometer is much narrower than the exit slit width *and* the exit slit bandwidth (slitwidth × spectrometer reciprocal linear dispersion) is much larger than the spectral line bandwidth, then under these conditions the spectrometer integrates the line spectral radiance to give an integrated radiance. Care has to be taken that the other nearby spectal lines from the emitting medium do not interfere with the measurement. The second way is to have the entrance slit of the spectrometer wider than the bandwidth of the source spectral line so at the exit focal plane the spectral line is integrated. Therefore, the exit slit width should have a bandwidth that is the same or smaller than the entrance slit. Again interference from other spectral lines is a potential problem. In either of these cases the narrow bandwidth of the spectral line is integrated so it can be compared to the continuum standard source. The bandwidth associated with this comparison is the convolution of the entrance and exit slit bandwidths. If the spectral line bandwidth is larger than that of the spectrometer, then a point-by-point wavelength scan of the spectral line must be accomplished and the spectral radiance can be determined by a direct comparison with the standard.

The integrated radiance in any of these cases is determined by integrating the calibrated spectral line data, so the signal from the continuum standard source can be compared directly to the signal from the observation of the integrated spectral line in the following way:

$$L_{\text{spec}}=L_{\lambda ,\text{lamp}}\;\Delta \lambda \;Signal_{\text{spec}}/Signal_{\text{lamp}}.$$ (7)

In all the above cases the resultant integrated radiance of the spectral line is obtained from Eq. (7), where Δ*λ* is the spectral bandwidth of the standard source integrated by the spectrometer’s bandwidth. This multiplication by Δ*λ* is necessary because the continuum emission of the calibrated source is given in units such as watts per cm^2^ per steradian per nm, while the spectral line is usually integrated in some fashion by the spectrometer, so its units are watts per cm^2^ per steradian. In the case where the power density in watts per cm^3^ is required, e.g., to compare with model calculations, the realization of this quantity can be accomplished only if the volume of the emission is known. To obtain the power per unit volume of the emission, *L*_spec_ of Eq. (7) must be multiplied by the ratio of the observed area of the surface of the standard emitting source (the spectrometer slit width × slit height × optical magnification) divided by the plasma volume viewed by the same optical system. In the simplest case this is just the volume defined by the cone of the slit being imaged through the emitting medium. This is not however a well defined quantity because of the possibility of optical aberrations and complex geometry [11]. It is just for this reason that the volume emission from a spectral line is calibrated as an equivalent surface radiator utilizing a surface emitting standard source integrated over the wavelength-band of the detecting system.

## 3. Experimental

This section deals with the specific equipment that has been utilized with the various reference cells to obtain current OES data.

### 3.1 Optical Equipment

The optical setups used in various reference cell OES experiments presented here [12–15] consist of an initial optical system, either lenses or mirrors, a spectrometer and a detector system, including computer control.

**Fig. 1:**
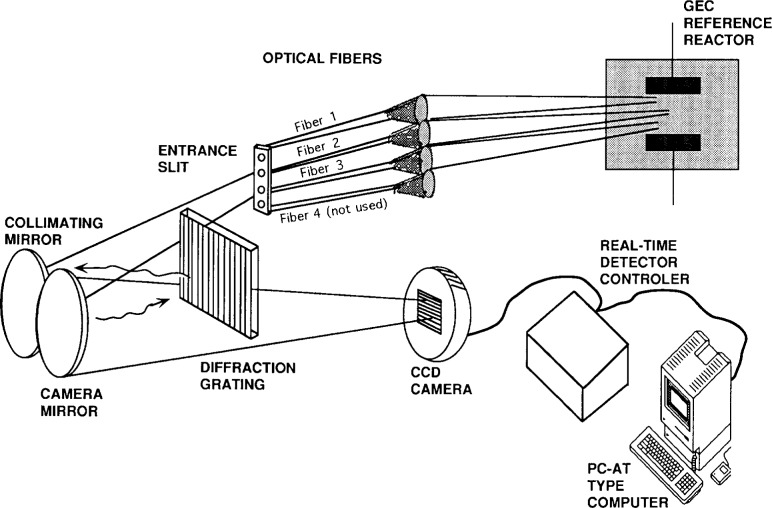
Schematic diagram of optical layout of Ref. [12].

The optics utilized in Ref. [12] and depicted in Fig. 1 are comprised of three quartz lenses used to focus the radial center of the electrode gap of the reference cell emission onto three 200 μm diameter, 1 m long quartz fibers. Lens-fiber 1 is focused approximately 4 mm from the upper electrode, lens-fiber 3 is focused approximately 4 mm from the lower electrode and lens-fiber 2 is focused at the center between the two electrodes. The fibers direct the emission from the plasma into a solid angle matching optical interface located at the entrance slit of the grating spectrometer. A torodial mirror grating spectrometer is utilized as the dispersive device. In this manner the spectra from three axial locations between the reference electrodes are observed.

**Fig. 2:**
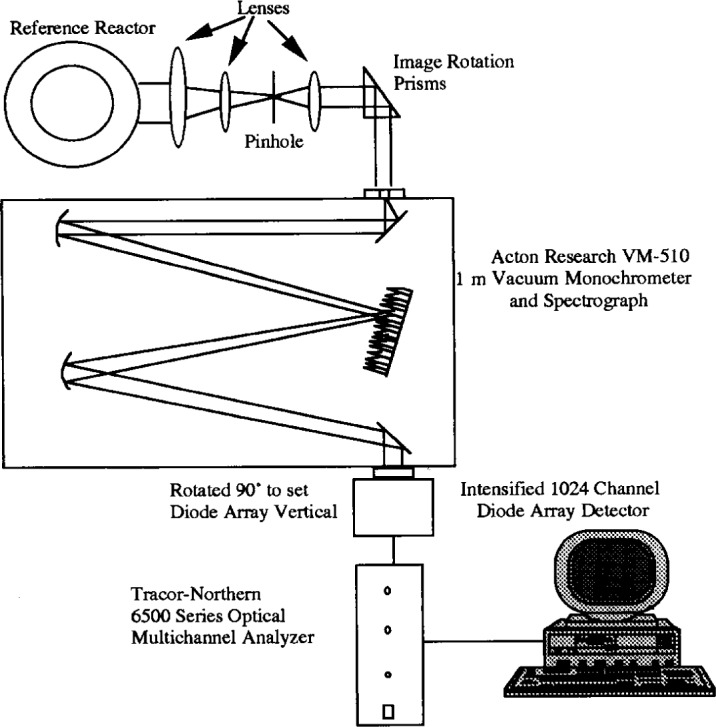
Schematic diagram of optical layout^1^ of Ref. [13].

The optics used in the experiments of Ref. [13] shown in Fig. 2 consist a series of lenses and a 500 μm diameter pinhole to image only parallel light from the emission between the cell electrodes with an image reduction of approximately 1/6. A prism is utilized to rotate the image, thus allowing a narrow horizontal sheet of radiation to be imaged onto the spectrometer entrance slit. The spectrometer used is a stigmatic 1 m focal length scanning vacuum monochromator-spectrograph which imaged the emission. Because of the parallel rays and the stigmatic spectrometer, the emission from the reference cell was imaged across its diameter at one wavelength. The optics were translated vertically to collect a two-dimensional view of the emission at one wavelength.

**Fig. 3:**
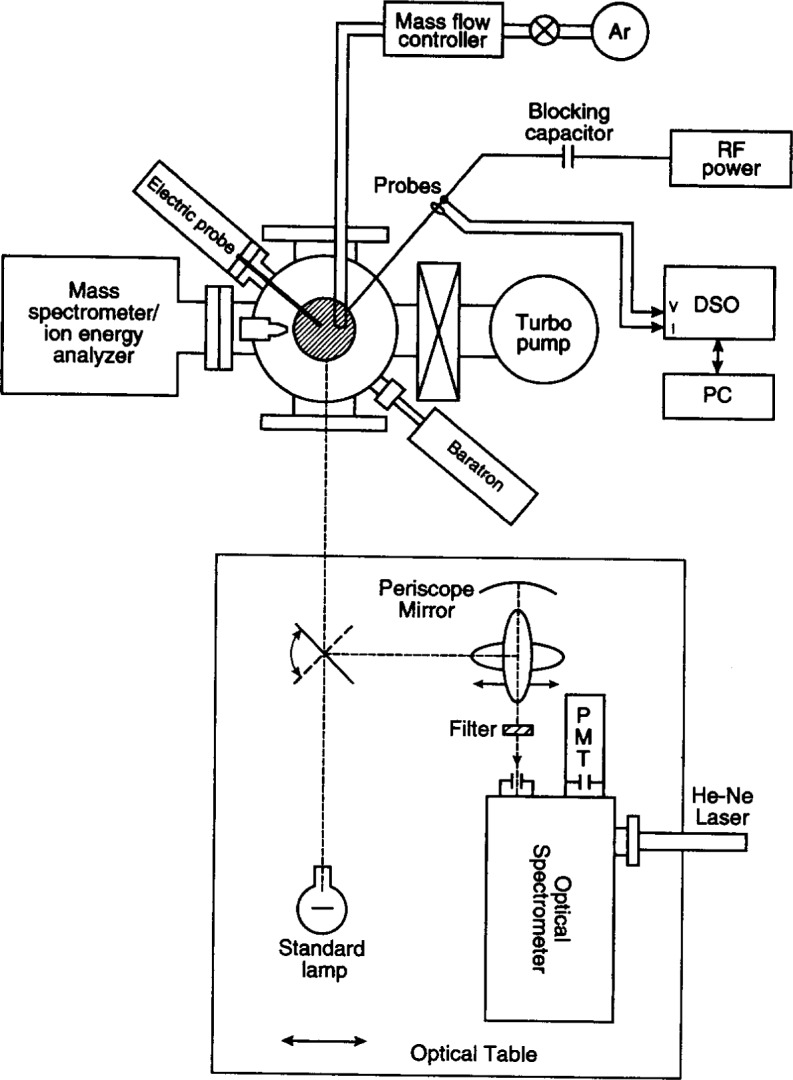
Schematic diagram of optical layout of Ref. [14].

The optics to image the plasma onto the monochromator slit in Ref. [14] (see Fig. 3) are front surface mirrors. A concave mirror is positioned so that the plasma image is demagnified onto the entrance slit by approximately a factor of 2. The mirrors are arranged to act as a periscope bringing the level of the plasma emission to the same height as the monochromator, as well as rotating the image of the plasma by 908. By this rotation, the electrode surfaces are imaged parallel to the long dimension of the entrance slit, thus permitting observations close to the surface and providing higher spatial resolution of the plasma along the vertical axis. The spatial resolution was 0.5 mm vertically and 4 mm horizontally. Because of the periscope, scanning of the plasma emission between the electrodes can be accomplished by translating one of the mirrors (see Fig. 3). The scans of the emission radial cross section are accomplished by a computer controlled, motorized translation of the entire optical system with respect to the stationary reference cell. The spectrometer is 1 m in focal length with parabolic mirrors and a quartz prism predispersor. A tungsten ribbon filament lamp calibrated for spectral radiance (± 2 % uncertainty) by the Radiometric Physics Division at NIST [10], is mounted on the optics table and is substituted for the plasma source by rotating one of the flat mirrors to image the lamp filament onto the slit (see Fig. 3). This lamp is used to calibrate the optics-monochromator system to obtain absolute spectral radiance measurements.

The optical system of Ref. [15] is not described in detail.

### 3.2 Detector Systems

The detector systems for Ref. [12] and Ref. [13] utilized array detectors to get spatial resolved OES profiles. The arrangement of Ref. [12] permitted the dispersed, wide bandwidth (~300 nm) spectral data to be obtained from three different positions between the electrode, depending on where each fiber optic was set to view (see Fig. 1). The array detector system in Ref. [13] was arranged in such a way that a horizontal radial profile could be gotten in a narrow wavelength bandwidth, and the imaging optical system was moved vertically to different portions of the axial profile (see Fig. 2), allowing a two-dimensional view of the emission.

A photomultiplier with associated electronic equipment comprised the detector system of Ref. [15].

**Fig. 4:**
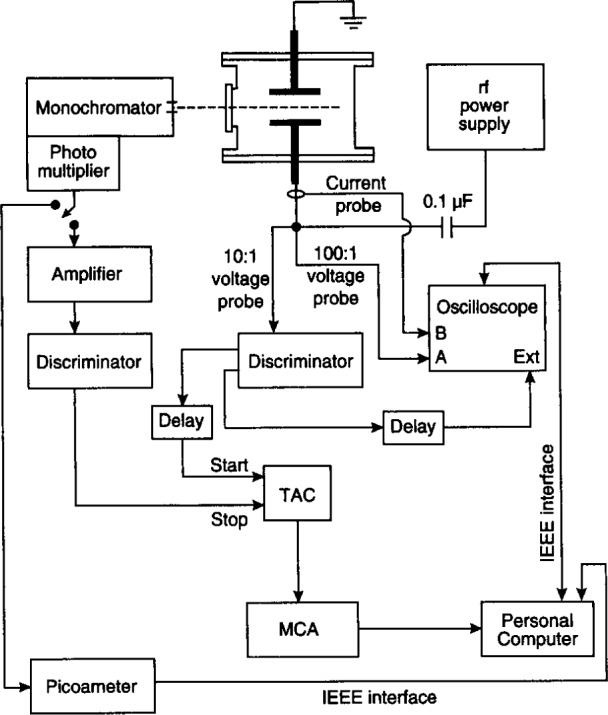
Schematic diagram of the detector and data acquisition system of Ref. [14], showing the two modes of data acquisition.

The detector system of Ref. [14] consisted of a photomultiplier and was operated in two modes. A detailed schematic diagram depicting these modes is shown in Fig. 4. In mode 1, the output from the photomultiplier was coupled to a digital pico-ammeter, which in turn sent the digitized signal to a PC based acquisition system to provide time-integrated data. The second mode is discussed in the section on temporally resoled OES and involved recording the time-varying output from the photomultiplier used in single photon counting with a system utilizing a time-to-amplitude converter (TAC) and a multi-channel analyzer (MCA).

## 4. Spatially Resolved OES Experimental Results

There are two different types of spatially resolved observations made on the reference cells. Since the configuration of the cell is cylindrical, one type is in the axial direction between the electrodes, while the other is a radial scan. The data obtained from both these types of scans are line-of-sight OES measurements. The deconvolution from line-of-sight or line-integrated (*x*,*y*,*z*) data to point-specific or radial distribution (*r*, *θ*) data is discussed in this section. The axial OES measurements are presented here without any deconvolution procedure.

### 4.1 Experimental Techniques

Radial distributions were accomplished in general by two different methods. One utilized translating the entire detector system, including the spectrometer, with a computer controlled motorized system. This was accomplished in Ref. [14] and Ref. [16] where the current mode, described in the section on detector systems, was used in the OES scans across the diameter capturing the line-integrated spatial distribution of the emission with a 0.5 mm vertical and 4 mm horizontal resolution. It was noted in these observations that the plasma extends well beyond the 10.2 cm diameter of the electrodes, showing the current flow from the powered electrode to the ground shields and the chamber walls as shown by the electrical measurements of Ref. [17]. In order to convert this line-integrated observations into a radial distribution, the data had to be Abel inverted [1]. The other method [13,18] to determine the radial optical emission utilized a diode array detector where a thin slice of the horizontal plasma emission was stigmatically imaged onto a 1m spectrometer slit and the optical imaging system was moved from point to point along the vertical axial emission, thus capturing both vertical and horizontal line-of-sight measurements. In Ref. [12], the spectra were recorded by the two dimensional CCD array for three different axial positions of the cell emission. No specific radial data was obtained from this experiment, only vertical axial information.

### 4.2 Abel Inversion

**Fig. 5:**
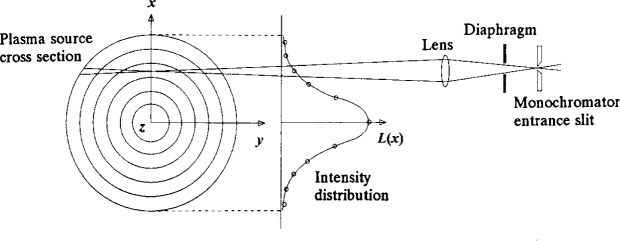
Diagrammatic representation of Abel inversion geometry for observations of plasma sources with cylindrical cross section.

Plasmas of cylindrical or near cylindrical symmetry that emit radiation that are observed along a chordal line-of-sight must have the cylindrical measurement transformed from the two dimensional *x*-*y* plane to the two dimensional *r*-*θ* plane in order to interpret the radial distribution of its properties. The process by which this is accomplished is called the Abel inversion. The mathematical formulation of this process is covered in many text books, e.g., Ref. [1], and is summarized as follows. If the plasma emission, *L*, is integrated along the *y* direction for a distance *x* (see Fig. 5) then an equation can be written to express this line-integrated emission in terms of the radial distribution of this emission as follows:

$$L\left(x\right)\;\Delta x\;\Delta z=\Delta x\;\Delta z\int_{-y}^{y}\varepsilon \left(r\right)dy$$ (8)

where Δ*x* is the observation length, Δ*z* is the plasma layer thickness, which is determined by the experimental aperture, e.g., the spectrometer slit height and width, and*ε*(*r*) is the local plasma emissivity at a distance *r* from the center of the cylinder. There are some assumptions that are usually made in reducing the complexity of this problem. The first assumption is that the variation in emission, d*L*(*z*)/d*z*, along the cylindrical axis direction, *z*, is small for the length Δ*z*. A second assumption defines the value for *ε*(*r*) = 0 for *r* ≥ *R*. Another assumption often made is that the plasma is symmetric so *L*(− *x*) = *L*(*x*), so Eq. (8) can be written as

$$L\left(x\right)=2\int_{0}^{y}\varepsilon \left(r\right)dy.$$ (9)

Using the substitution *r*^2^ = *x*^2^ + *y*^2^, Eq. (9) becomes

$$L\left(x\right)=2\int_{x}^{R}\frac{\varepsilon \left(r\right)r\;dr}{\sqrt{r^{2}-x^{2}}}.$$ (10)

This is a special form of the Abel integral equation and if *L*(*x*) = 0 for all *r* ≥ *R*, Eq. 10 can be inverted analytically [1] into

$$\varepsilon \left(r\right)=-1/\pi \int_{r}^{R}\frac{L\prime \left(x\right)\;dx}{\sqrt{x^{2}-r^{2}}}$$ (11)

where *L*′(*x*) = d*L*(*x*)/d*x*. If the emission is not symmetric, another approach must be used [19].

In the experiments on the reference cell, *L*(*x*) is a set of time-integrated values measured at discrete values of *x*. If *L*(*x*) is in a functional form, Eq. (11) can be solved analytically, if not, the integral must be solved numerically. In practice the measured values of *L*(*x*) are approximated by an analytical function or set of functions, symmetric or non-symmetric. If any method presupposes d*L*(*x* = 0)/d*x* = 0 because of plasma symmetry, then this must be an additional constraint in the Abel inversion method and only symmetric functions should be used.

There have been a number of techniques developed to solve the Abel integral equation, some graphical, some numerical and others analytical. Using a polynomial least squares fitting procedure for the experimental data, *L*(*x*), to produce an analytical inversion was suggested by Ref. [20]. Similar methods were used by Ref. [21], Ref. [22], and Ref. [23]. Another method employing sets of orthogonal polynomials of arbitrary order have been used for the analytical fit. Examples of such solutions include Zernike polynomials in Ref. [24], Gegenbauer polynomials in Ref. [25], Hermite polynomials in Ref. [26], Gram polynomials in Ref. [27], and a general set of orthogonal polynomials in Ref. [28]. Spline fits have also been popular to use to fit the experimental data and then perform the Abel inversion process. Authors employing such methods include Ref. [29], Ref. [30], Ref. [31], Ref. [32], Ref. [33], and Ref. [34]. Another recently developed method has been the use of fast Fourier transforms to perform the Abel inversion [35]. This is particularly appealing when the transformed data shows a distinct separation of low frequency components associated with the real spatial emission distribution and high frequency components that can be assigned to experimental noise or plasma fluctuations. If this distinction does not occur in the transformed data, this method is equivalent to the others in performing an Abel inversion of the experimental data. There are many more methods that have been used, but their premisses are essentially the same as the ones already listed.

Noise and fluctuations can present a problem in the Abel inversion process. In the fast Fourier transform method, a filtering algorithm can be applied to take out the unwanted components if they can be identified. One can choose smoothing factors associated with analytical fitting methods as well, but it is particularly difficult to know what order of polynomial to choose to do this to have the most confidence in the Abel inversion process. For example, in the reference cell there exists plasma radially outside the dimensions of the cylinder defined by the electrode diameter. Under certain conditions of pressure and voltage, this requires scanning the plasma emission all the way to the maximum of the observation window diameter. Observation at the window edge is complicate by emission entering the observation acceptance solid angle through reflections off the walls showing a “bump” in the plasma emission that may not be real. Making the solid angle of the observation smaller may allow one to approximate the true emission distribution outside the electrode diameter, but this reduces the signal as well. Another observed fluctuation in the emission that can interfere with the inversion procedure is the effect of the observation window’s surfaces acting as a low finesse interferometer. This effect has been observed by eye in the reference cell as well as in the scans of the emission. It results in a wavelength dependent modulation on the optical emission signal and will be exhibited in its Abel inverted radial distribution. The effect can be eliminated by increasing the spectrometer bandwidth by opening one of the slits.

The Abel inversion algorithms require a zero value along the scan axis of the function to be inverted. Often this has to be artificially produced. In the case of the reference cell and most radially distributed plasma emission, this problem is not serious, since the magnitude and length of the emission is small compared to the maximum emission. However, it can be a problem if the plasma emission has a substantial dip in its central region. The absolute magnitude of the dip in the emission will have a larger uncertainty under these circumstances.

Another concern in the Abel inversion process is the possible absorption of the emission under observation, i.e., an optical depth problem. This is of general concern in discharges especially involving optical transitions where the lower state is the ground state or a metastable state. Thus, emission from these type of transitions must be checked for optical depth to determine if this is a problem. In the section on fundamental concepts, the radiative transfer equation [Eq. (1)] addressed this problem with a term corresponding to reduction of the emission. As suggested in this section, the optical depth can be tested for and actually its effect observed by placing a concave mirror located along the line-of-sight of the observation at a distance equal to twice its focal length. This in effect focuses the emission back onto itself. The correction for the optical depth can be included in the Abel inversion process but will not be discussed here. This procedure has been treated by Ref. [1] and Ref. [20].

### 4.3 Experimental Results

#### 4.3.1 Comparison of Radial Abel Inverted OES and LIF Measurements

**Fig. 6:**
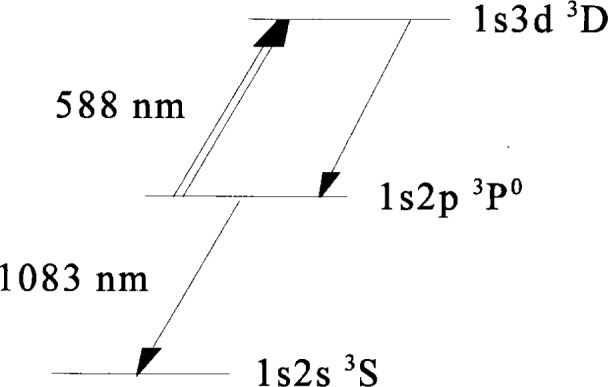
Partial energy level diagram showing the LIF pumping wavelength at 588 nm and the OES from 588 nm and 1083 nm.

**Fig. 7:**
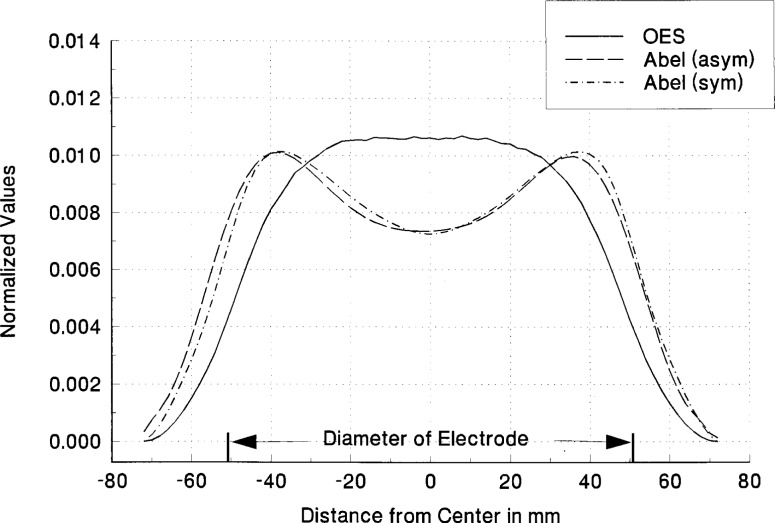
Comparison of two different Abel inversion techniques. Abel (asym) (– – –) represents an algorithm that fits the OES data with a 8th order Chebyshev polynomial, but does not assume radial symmetry at the electrode center. Abel (sym) (·−·−·−) represents an algorithm which symmetrizes the OES data (— – —) and defines a new emission center. The normalized OES signal is also shown for comparison. All data were obtained in a He discharge at *P* = 67 Pa and an applied peak-to-peak rf voltage (*V*_rf_) of 200 V.

**Fig. 8:**
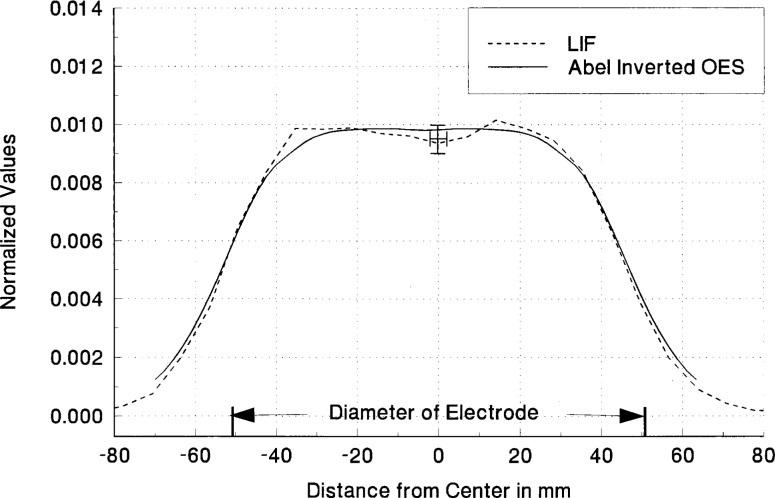
Comparison of the radial distribution of the emission from the spatially resolved LIF measurements at 588 nm (– – –) and the Abel inverted OES at 1083 nm (— —) using the asymmetric algorithm obtained in a He discharge at *P* = 67 Pa and a *V*_rf_ = 200 V. The OES Abel inversion process included the fact that the 1083 nm line was corrected for optical depth. These radial distributions are.

For comparison of the Abel inversion process, two different methods are used as examples to provide radial emission distributions for some conditions in the reference cell. These Abel inverted radial profiles are also compared with radial distribution data at the same conditions obtained from the laser induced fluorescence (LIF) method. The experiment was performed in helium because of its simple spectrum [36]. Also the comparison had to be made using the same atomic level that was probed by both methods. Therefore, the following scheme was chosen (see Fig. 6). For this experiment the LIF was performed using a wavelength of 588 nm to pump the electron-collisionally populated 1*s*2*p* ^3^P^o^ lower state to the 1*s*3*d* ^3^D upper state and observing the fluorescence at 588 nm. The OES was done by observing the optical transition from the 1*s*2*p* ^3^P^o^ to the 1*s*2*s* ^3^S metastable state at 1083 nm. The two methods for the Abel inversion comparison use 1) the Chebyshev polynomial of 8th order to fit the experimental data and then the inversion of this analytical function and 2) a spline fit to the experimental data with an averaging method to symmetrize the experimental profile before Abel inversion. These two methods were equivalent and showed no significant differences outside the experimental fluctuations as seen in Fig. 7. In the application of the Abel inversion, there had to be a small correction for the optical depth of the 1083 nm emission line. This correction was accomplished by the method outlined above using a concave mirror which is translated as the spectrometer-detector system scanned across the plasma diameter and a convolution method outlined in Ref. [1]. This correction was small for the Abel inversion and the comparison between this method and the LIF data shows agreement well within the experimental uncertainty as shown in Fig. 8. In this experiment levels were chosen to compare Abel inverted OES and LIF so that there was a common level to be probed, i.e., the lower level for the absorption in the LIF was the same level a the upper state for the optical emission. The error bar in Fig. 8 in the horizontal direction represent the minimum spatial resolution of the overlap of the optical system and the laser beam, while the vertical error bar represents an estimate of the uncertainty (1 s) from the fluctuations in the data due to the non-reproducibility of the laser’s output.

#### 4.3.2 Correlation of OES With Etching Processes

Abel inverted radial profiles of the optical emission in Ar and Ar + CF_4_ have also been observed in the reference cell [18]. The discharges were investigated at the different reference conditions and the OES from the 750 nm Ar I and 428 nm Ar II lines were recorded. The optical emission from the reference cell was viewed across the diameter of the discharge, parallel to the electrodes and at various heights between the electrodes. The method of analysis utilized a polynomial fit to the OES data and the subsequent inversion of this analytical function to perform the Abel inversion. The profiles of the OES were found to extend radially well beyond the electrodes for certain plasma conditions. The explanation for this was postulated to be due to the cylindrical ground shields that surround the electrode insulators. These shields were originally included in the design of the reference cell when preliminary experiments showed unstable plasmas without them. A particle-in-cell code was applied to this region and it predicted a strong effect that drove high energy electrons thus creating a nonuniform electric field to increase the excitation of argon near the powered electrode region. These profiles were compared to radial distribution of etch rates of a silicon wafer. The etching was accomplished in a plasma with 30 cm^3^ per minute flow of CF_4_ and 76 mm diameter silicon wafers. The wafers were patterned prior to etching to enable the etch rate to be determined. The optical emission from the reference cell was viewed across the diameter of the discharge, parallel to the electrodes and at various heights between the electrodes.

**Fig. 9:**
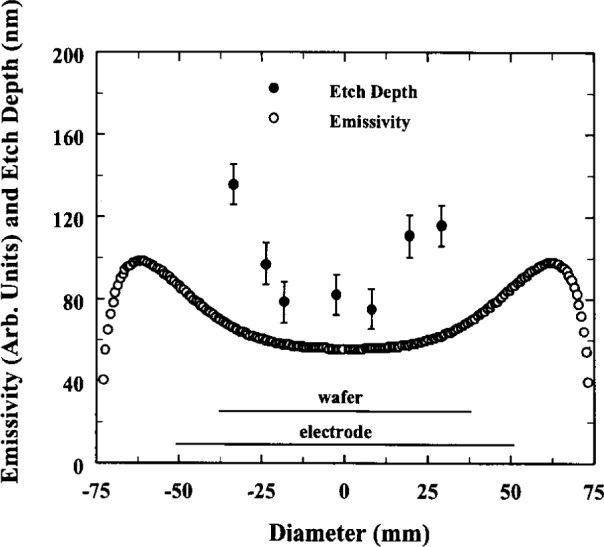
Comparison of etch depth in patterned silicon and Abel inverted OES from Ref. [37].

Radial etch distributions were also investigated with OES by using Ar + CF_4_ and Ar + CF_4_ + O_2_ etching plasmas under various conditions [13,37]. The OES profiles were compared to radial distributions of etch rates of silicon wafers. The wafers were patterned prior to etching to facilitate the measurement of etch uniformity. The results are shown in Fig. 9 where the nonuniform increasing etch rate away from the center of the discharge corresponds to the Abel inverted OES profile. This distribution in the radial etch rate, shown by the points in Fig. 9, was observed with both a 76 mm Si wafer directly on the aluminum electrode as well as a similar wafer separated from the electrode by a quartz plate. Since the etch rate was the same both with and without the quartz plate, this indicates that the etch rate was not due to a fluorine loading effect in the plasma.

In Ref. [12] 102 mm patterned silicon wafers with 830 nm of oxide were etched with a CF_4_-CHF_3_ discharge. Etch rates from 8.5 nm/min to 60 nm/min were observed depending on the operating parameters. OES was performed using the fiber optic imaging system of Ref. [12] and the oxide etch rate was compared to the univariate correlation as a function of the spectral data from 178 nm to 482 nm. The spectral range from 190 nm to 300 nm was noted to show the largest variance and this was associated with the CF*_x_* etchant species whereas this spectral range showed the lowest univariance correlation to the oxide etch rate. These experiments indicate that OES can be used in multivariate analysis to predict oxide etch rates.

In Ref. [15] the OES from the discharge containing reactant gases was used to monitor the etching endpoint. This was accomplished by recording the OES of reactant gases without the presence of a Si wafer in the cell. Then the same spectrum was recorded with a wafer present. The two spectra were compared and the change in peak signals of reactant gas spectral line as well as the peak signal in etchant product spectral line were recorded. Three different etching processes investigated in this manner were, 1) photoresist stripping with a pure oxygen plasma, 2) silicon etching with a SiF_6_-O_2_ mixture, and SiO_2_ etching with a Freon 14-H_2_ mixture. These investigations also included selectivity and etch rate ratios of common etchant processes for oxide and polysilicon. Wafers coated with 400 nm of polysilicon over 100 nm of oxide were used. The polysilicon and oxide were exposed alternately across the wafer using conventional lithographic methods. Then a layer of photoresist was patterned onto the wafer such that all three films (oxide, polysilicon and photoresist) are exposed. The patterned wafer is then placed into the reference cell and about 25 % of the film is removed. The thicknesses of all films are then measured and the etch rate and uniformity are determined. This process is repeated until the film is nearly or completely removed and the selectivities and etch rate ratios are thereby determined.

#### 4.3.3 Axial Scans of OES

One of the problems in understanding rf discharges has always been the identification of the basic mechanism that sustains the discharge. Some discharges are maintained by stochastic heating of the electrons at the sheath boundary. Under certain conditions most of the ionization in the plasma comes from the electrons created by ion impact on the electrode. These electrons are multiplied by ionizing the gas in the glow region at the edge of the sheath where they are accelerated in the electric field of the sheath. These and other different sources of energetic electrons, ions and atoms lead to different shapes of the spatial profile of emitted radiation for different species and correspondingly of the ionization. Therefore, the spatial profiles have been used to gain some understanding of the sustaining mechanisms in many rf discharges.

**Fig. 10:**
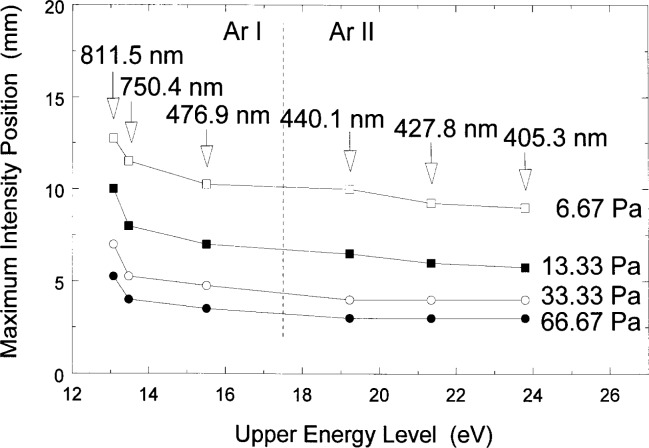
Plots of the maximum intensity position of various spectral line of Ar I and Ar II as measured from the powered electrode as a function of the excitation energy of their OES upper states for different pressures.

OES from certain emission lines with the highest excitation energy, i.e., a higher threshold for excitation, have been noted to appear closer to the electrodes when electrons are accelerated in the sheath and make a significant contribution to excitation [14]. When this is not the case, it is also expected that the lines with lower threshold energies will occur over a broader spatial region around the sheath boundary where electrons are accelerated. In a recent experimental study [38] results of spatially resolved optical emission of neutral and ionic argon lines from pure argon rf discharge along the axial direction between the electrodes in the reference cell show the spatial distribution of emission where the lines with highest upper energy level appear closest to the electrode (see Fig. 10). Since the emission distribution is used in experiments to determine the sheath kinetics, it is important to understand why the spatially resolved profiles of different ions have such different shapes and positions of their spatial maximum emission. For example, some diagnostic techniques, such as actinometry, should be used with caution because of the different spatial distributions of the lines used for this technique.

## 5. Temporally Resolved OES Experimental Results

The method of temporally resolving OES data on the time scale of the 13.56 MHz rf frequency applied to the reference cell is either to measure the OES signal as a function of time over one or more periods (73.75 ns) or to take a snapshot of the temporally resolved OES signal [39]. The first method requires a detection-acquisition apparatus capable of taking multiple observations over the time interval selected. Such an apparatus is described in the section on detector systems. In the snap shot method, for an apparatus to provide real temporal resolution, the time-aperture must be short compared to the OES signal variations over one rf cycle. None of the experiments reported here used this later technique.

### 5.1 Temporally Resolved OES in Ar I and Ar II Discharges

The multiple point observation method over at least one period of the rf cycle was the only method used to temporally resolve OES in the reference cell. In mode 2 of Ref. [14], the time-varying output from the photomultiplier used in single photon counting was recorded with a system utilizing a time-to-amplitude converter (TAC) and a multi-channel analyzer (MCA). A detailed schematic diagram depicting this mode of data acquisition is shown in Fig. 4. The timing cycle of the TAC is initiated by a fast pulse derived by a discriminator from a probe attached to the bottom of the powered electrode. The timing cycle is stopped by a photon-initiated pulse from the photomultiplier system, or by the ending of the preset TAC timing period. The maximum time measured by the TAC was set to 200 ns (slightly less than 3 rf cycles). The photon count rates were kept low (< 10^5^ s^−1^) by placing calibrated neutral density filters in the optical path for the measurement of the most intense optical signals. On average fewer than one photon is detected for each timing cycle (< 10^5^ s^−1^ × 74 ns ⟹ < 7.4 × 10^−3^ counts/cycle), therefore, there is no discrimination against photons detected later in the timing cycle.

The output pulses from the TAC, whose voltage amplitudes are proportional to the time between the trigger from the rf waveform and the detection of the photon, are sorted into channels by the MCA. This accumulated spectrum represents the time-resolved optical emission signal for a single spatial location. The MCA was set to record 256 channels providing a timing resolution of 0.78 ns for each channel.

The radiometrically calibrated temporally resolved optical emission from Ar I, 750.4 nm and Ar II, 434.8 nm is presented in Ref. [14] for all of the reference cell reference conditions.^2^ This was accomplished over approximately two rf cycles (147.5 ns) and for the space between the electrodes along the central axis. These two spectral lines were chosen because they were among the strongest signals and their upper state atomic lifetimes were among the shortest, thus allowing the maximum flexibility in observing the temporal evolution of the optical emission. These radiometrically calibrated results under reference conditions allow cell-to-cell and model-to-cell comparisons.

### 5.2 Determination of Velocity Distributions

**Fig. 11:**
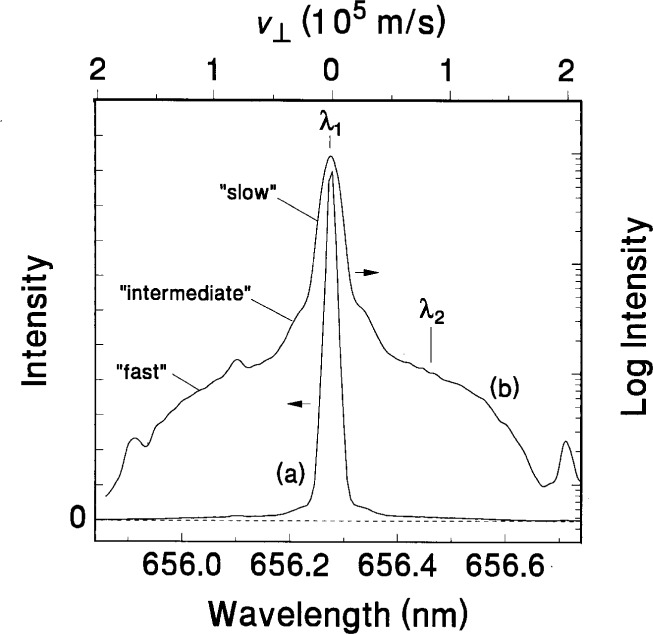
Linear (a) and semi-log (b) plots of the H_α_ spectral line profile emission for a 33.3 Pa, *V*_rf_ = 350 V hydrogen discharge, demonstrating the three velocity components of the hydrogen atoms in the *n* = 3 state. The points *λ*_1_ and *λ*_2_ indicate the wavelengths at which the temporal resolved data presented in Fig. 12 were obtained. The upper scale indicates the perpendicular velocity component to the Doppler shift, Δ*λ*, from the line center at 656.3 nm.

**Fig. 12:**
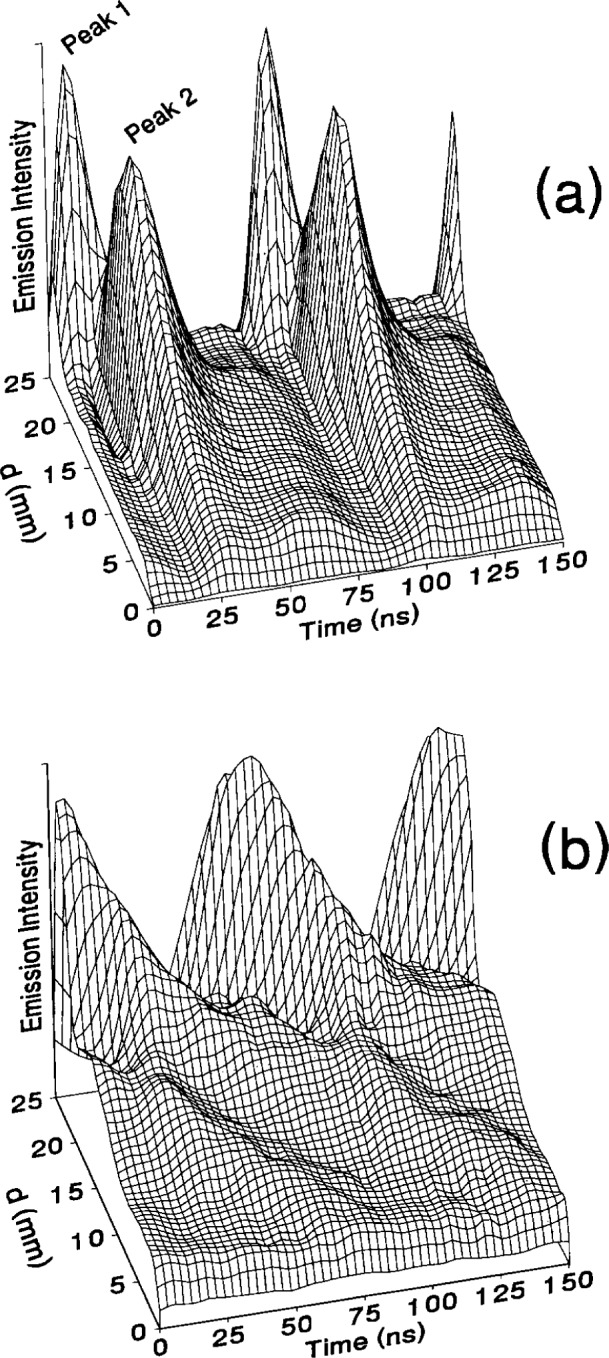
Three-dimensional surface plots of the temporally and spatially resolved emission of H_α_ as measured from the grounded electrode. (a) and (b) are the plots for *λ*_1_ and *λ*_2_, respectively as defined from Fig. 11. Each plot is normalized to its maximum signal.

The evidence of multiple velocity distributions of atomic species in the reference cell has been investigated using hydrogen and hydrogen-argon mixtures plasmas. The methods of investigation include OES [40,41] and ion-energy mass spectrometry (IEMS) in conjunction with OES measurements [42]. By investigating the spectral line profiles of hydrogen Balmer lines, H_α_ and H_β_ [40] unique effects on the shape of these line profiles were associated with hydrogen atoms with distinct velocity distributions strongly effected by admixtures of argon. Similar effects have been observed in discharges in pure H_2_ discharge in the reference cell [41]. Here the H_α_ profile showed three distinct velocity distributions, one associated with the bulk low temperature plasma, a second, very small concentration associated with an intermediate temperature, and a third velocity distribution correlated to very high energies (see Fig. 11). These velocity distributions were correlated with spatially and temporally resolved OES (see Fig. 12). The first was correlated with the excitation of H atoms at the beginning of the cycle of the rf power due to dissociative electron-impact excitation of thermal H_2_ resulting in H atoms with thermal energies < 0.2 eV, while the intermediate component is due to dissociative ionization of H_2_. The third component was associated with H atoms streaming across the interelectrode space at high velocity far exceeding energies possible from electron-impact. The fast H atoms are formed near the powered electrode and are detected throughout the entire plasma volume with kinetic energies in excess of 100 eV. These measurements also indicate that the velocity distribution of H atoms is anisotropic, with the highest velocity component along the axis of the electrodes, in contrast to the velocity perpendicular to this axial direction. Temporally resolved measurements indicate that the fast H atoms are a result of backscattering of neutral H from the surface of the powered electrode due to bombardment of that electrode surface by fast H ions and neutrals formed in the discharge. Discharges with volume fraction mixtures of 90 % argon and 10 % hydrogen have also been investigated [42] by spatially and temporally resolved OES and mass-resolved measurement of ion kinetic energy distributions at the grounded electrode. By measuring kinetic energies of mass-identified ions striking the grounded electrode of the reference cell it was possible to determine the dominant ion species present in the discharge and to infer the most likely ion formation processes for each species.

Another phenomenon observed in studies of the optical emission from rf discharges in hydrogen is the presence of a “double sheath.” This has been observed previously in rf discharges for time averaged [43] and time resolved investigations [44]. The formation of these double sheaths have been attributed to the faster electron and ion drift in hydrogen, which contributes to the formation of an electric field in front of the powered electrode during the positive portion of the voltage waveform. This electric field allows the acceleration of electrons toward the powered electrode, which subsequently excite H_α_ emission and the formation of a second region of emission.

**Fig. 13:**
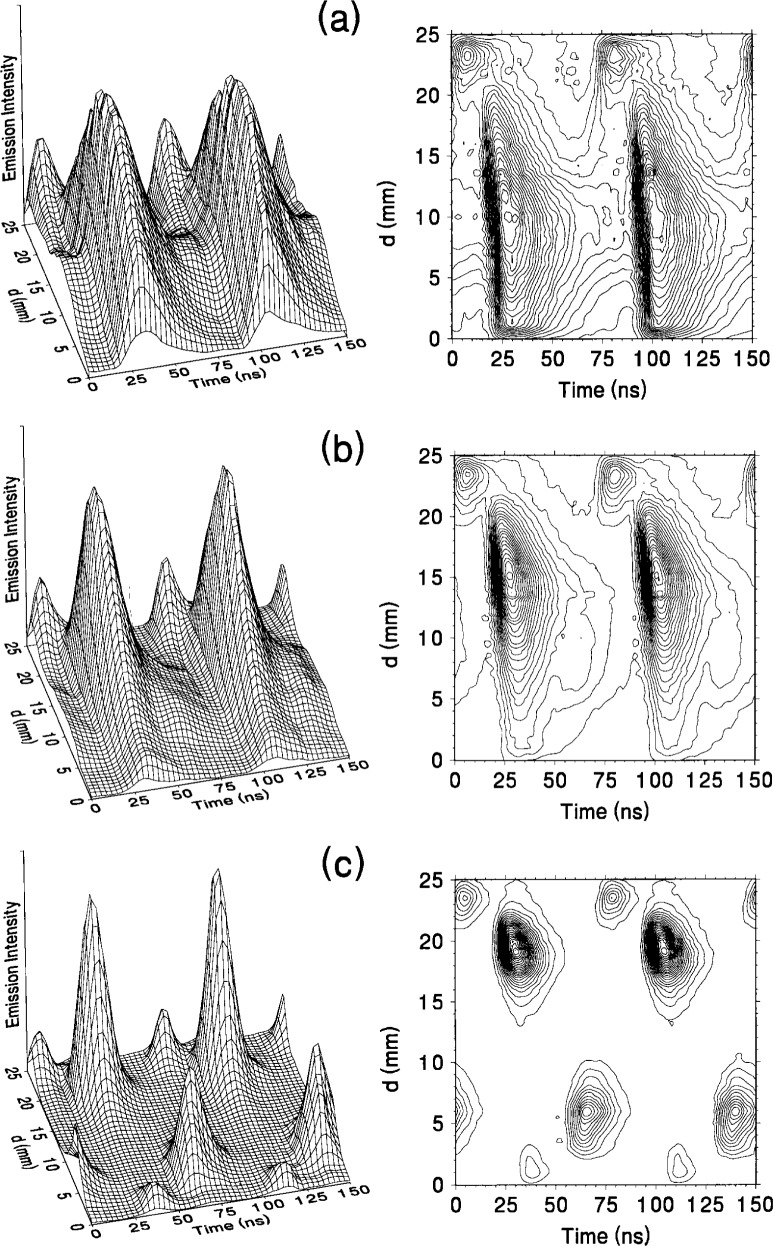
Three-dimensional surface and contour plots of the temporally and spatially resolved, double sheath emission of H_α_ as measured from the grounded electrode with *V*_rf_ = 200 V. (a) *P* = 13.3 Pa, (b) *P* = 33.3 Pa and (c) *P* = 133.3 Pa. The plots are from the wavelength position *λ*_1_ as defined from Fig. 11. Each plot is normalized to its maximum signal.

**Fig. 14:**
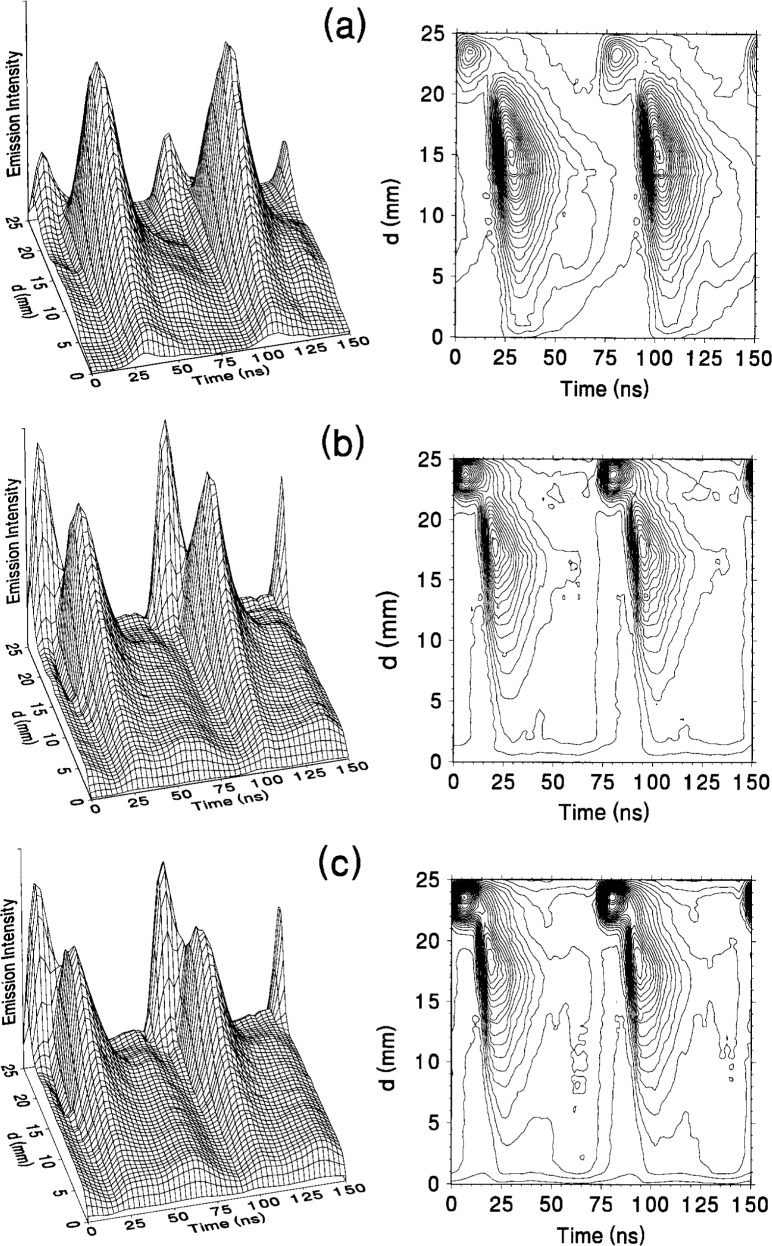
Three-dimensional surface and contour plots of the temporally and spatially resolved, double sheath emission of H_α_ as measured from the grounded electrode with *P* = 33.3 Pa. (a) *V*_rf_ = 200 V, (b) *V*_rf_ = 350 V and (c) *V*_rf_ = 450 V. The plots are from the wavelength position *λ*_1_ as defined from Fig. 11. Each plot is normalized to its maximum signal.

This double sheath behavior has also been investigated for hydrogen discharges generated in the GEC rf Reference Cell. The temporally and spatially resolved emission from hydrogen discharges as a function of pressure and voltage are presented in Figs. 13 and 14, respectively. The signal is shown for a time range of slightly more than two rf periods, the applied rf voltage peaks at time *t* = 0, and *d* is the distance from the grounded electrode.

The conditions for the data in Fig. 13a (*V*_rf_ = 200 V, *P* = 133.3 Pa) are nearly the same as the conditions for the data presented by Ref. [44], and the data are in agreement. The double sheath is evident by the pair of peaks that appear near the electrodes. At lower pressures (Fig. 13a) the peaks occur only near the powered electrode. The large peak is due to the normal excitation that is observed in an rf glow discharge, similar to that observed in argon [14]. The smaller peak that occurs closer to the powered electrode, and precedes the larger peak is the “second sheath” that is unique to hydrogen and electronegative gases. As the pressure increases the magnitude of the smaller peak remains constant, while the intensity of the larger “glow” peak increases. At 133.3 Pa, the discharge becomes sufficiently symmetric that the double sheath behavior is also observed near the surface of the grounded electrode (Fig. 13c).

As the rf voltage is increased for a constant pressure (see Fig. 14), the intensity of both peaks increases substantially, but the magnitude of the peak closest to the powered electrode increases much more dramatically by comparison. This is in agreement with the explanation of Ref. [44], since higher applied voltages will produce higher energy electrons in the sheath region during the positive portion of the rf cycle, and thus contribute to the emission of the “second sheath.”

## 6. Other Experiments Utilizing OES

Although the experiments described in Refs. [45] and [46] utilize the GEC rf Reference Cell as an experimental platform, they are performed with the cell in a modified configuration. However, OES was performed as a probe in these experiments, therefore, a summary of them is included here for completeness. In the experiments of Refs. [45] and [46] OES was utilized to detect and quantify the presence of “dust” particle traps in the plasma. The experiments are performed by observing the OES above a grove cut into a square Al plate, 10 cm on a side, placed on the cell’s lower electrode. The observations were cylindrical line-of-sight measurements, spatially resolved by collimating apertures. The OES was performed on the (420.1, 451.1, 592.9, and 604.3) nm Ar I lines and all observations showed the same behavior. The optical emission was found to vary spatially due the presence of the electrostatic traps formed by the grooves, thus their positions were quantified.

## 7. Concluding Comments

This review of the OES experiments utilizing the GEC rf Reference Cell, combined with the others within this volume, has demonstrated its use as versatile tool to compare experimental as well as model investigations with one another. It was found that the use of a multiple of different observational techniques can be correlated with one another. In the case of temporally resolved OES and IEMS investigations, a correlation has been found to uniquely describe three velocity components of H atoms and their origins. Also correlation between Abel inverted spatially resolved OES and etch rates in silicon has been demonstrated, thus providing a potential tool for possible etch uniformity measurements. Experiments have also been performed that indicate that OES can be used in multivariate analysis to predict oxide etch rates. Experiments with etching gases have also demonstrated the use of the reference cell as teaching tool.

## Footnotes

1 Certain commercial equipment, instruments, or materials are identified in this paper to foster understanding. Such identification does not imply recommendation or endorsement by the National Institute of Standards and Technology, nor does it imply that the materials or equipment identified are necessarily the best available for the purpose.

2 It should be noted here that the units used in Ref. [14] were given in units of W/cm^3^ (irradiance). The results of the calibration of these spectral lines did not include the spectral bandwidth. Therefore, the values of all the figures should be multiplied by 10^−8^ cm, the spectral bandwidth of the observation, to give the results in W/(cm^2^ · sr) (radiance).
